# A Synthetic Review of Feedbacks and Drivers of Shrub–Grass Interaction in the Process of Grassland Shrub Encroachment

**DOI:** 10.3390/plants14040605

**Published:** 2025-02-17

**Authors:** Huiyang Hou, Haoran Yan, Xue Bai, Yuzhen Zhang, Yanjun Guo, Jianwei Zhou, Shaobo Gao

**Affiliations:** 1 Grassland Research Institute of Chinese Academy of Agricultural Science, Hohhot 010010, China; houhuiyangg@163.com (H.H.); 15061707109@163.com (Y.Z.); yanjung2024@163.com (Y.G.); 2 College of Grassland Science, Qingdao Agricultural University, Qingdao 266109, China; 3 Innovation and Breeding Research Institute of Mengcao Group, Hohhot 011517, China; 4 Inner Mongolia Autonomous Region Agricultural Technology Extension Center, Hohhot 010010, China; nmgbaixue@163.com; 5 Institute of Grassland Research, Chinese Academy of Agricultural Sciences, Key Laboratory of Biohazard Monitoring and Green Prevention and Control in Artificial Grassland, Ministry of Agriculture and Rural Affairs, Hohhot 010010, China

**Keywords:** shrub–grass interaction, stress gradient hypothesis, shrub encroachment, community succession

## Abstract

Many grasslands around the world are affected by shrub encroachment. The essence of shrub encroachment into a grassland habitat is a change in the direction and intensity of shrub–grass interactions, which leads to an alteration in the grassland community structure. Recent research progress can be summarized as encompassing the primary factors influencing shrub encroachment and the physical, biological, and chemical ways through which they affect grassland community succession and shrub–grass interactions. The purpose of this study was to explore how shrub–grass interactions and relationships change under the influence of various environmental factors and their impact on grassland communities to provide a theoretical basis for grassland restoration and the management of shrubs within grassland from the perspective of shrub–grass interaction.

## 1. Introduction

Plant–plant interactions are generally considered to be one of the most important parts of the mechanism by which environmental change drivers affect species composition and the community structure [1]. Due to climate change and anthropogenic disturbances, the abundance of shrub species in grassland ecosystems is increasing and the phenomenon of grassland shrub encroachment is exacerbated [2]. The essence of grassland shrub encroachment can be understood as a change in the interaction between shrub and grass plant species, which alters the structure and appearance of the community. Shrub–grass interactions include positive (facilitation), negative (competition), and neutral interactions [3]. The balance between positive and negative interactions hinges on the intensity of abiotic stress [4]. This conceptual model of the “stress gradient hypothesis” (SGH) effectively predicts and reveals the patterns of plant–plant interactions in various ecosystems in response to abiotic stress gradients [4].

In recent years, there has been an increase in studies of interactions between shrubs and other plants [5,6]. Shrub–grass interactions are considered an important mechanism for maintaining ecosystem functions (such as biodiversity and productivity) in grasslands undergoing shrub encroachment [7]. The effects of shrub–grass interaction on communities can be modified by external drivers such as climatic conditions or biological disturbances. At present, although scholars have conducted comprehensive studies on the drivers of shrub encroachment [8,9], there are few studies examining shrub encroachment from the perspective of the environmental impact of shrub–grass interactions. The influence of the indirect shrub–grass interaction mechanism on shrub encroachment also needs to be further explored. There is no consensus regarding the mechanism of shrub–grass interactions and their impacts on community succession in the process of grassland shrub encroachment. Considering the important position of shrub–grass interaction and the current background of global climate change, it is of great significance to explore the mechanism of shrub–grass interaction and how the environmental change process influences it.

This review focuses on three aspects of recent advancements: the primary drivers; the occurrence and mechanisms of physical, chemical, and biological factors; and their influence on community succession and shrub–grass interaction. Based on recent advancements related to shrub–grass interaction, the problems and research directions that need to be further explored in future research are outlined and recommended. The purpose of this review is to explore the changing trends of shrub–grass interactions in the presence of various environmental factors and their influence on grassland communities to provide a reference for dynamic changes in shrub–grass presence and interaction.

## 2. Major Drivers Affecting Shrub–Grass Interactions

**Figure 1:**
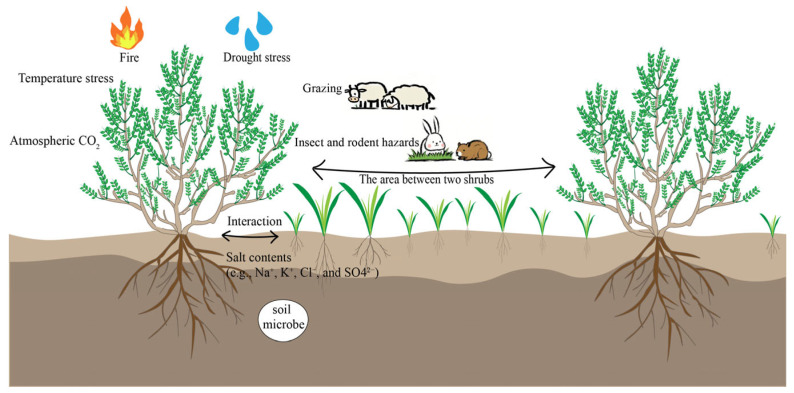
Factors affecting shrub–grass interaction.

**Figure 2:**
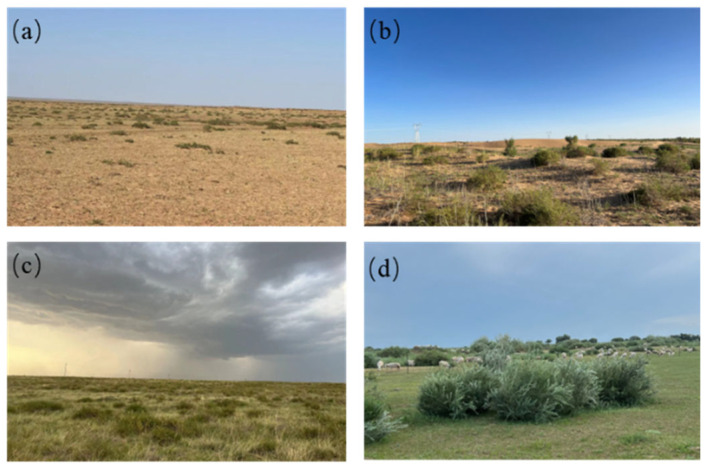
Examples of shrub-encroached grassland landscapes: (**a**) shrub-encroached grassland under drought stress; (**b**) shrub-encroached grassland after desertification; (**c**) shrub grassland under abundant precipitation; and (**d**) shrub grassland under grazing disturbance.

Shrub–grass interactions may be disturbed by biological and abiotic stresses such as grazing, drought, temperature, salinity, fire, and the atmospheric CO_2_ concentration in the survival environment (Figure 1 and Figure 2). According to the stress gradient hypothesis (SGH), the intensity of plant–plant interactions is closely related to the degree of stress and disturbance of the external environment in which the plant is located. The original hypothesis indicated that facilitation increases monotonically with the degree of environmental stress [10]. However, a multitude of empirical studies have shown that facilitation results in a unimodal pattern as environmental stress increases. When the degree of environmental stress is low, plants mostly experience competition. Under moderate stress, facilitation dominates, but under higher levels of stress, facilitation decreases or even changes to competition. The changing pattern of shrub–grass interactions under environmental stress provides an important theoretical basis for understanding the construction of shrub encroachment in grassland communities [11].

### 2.1. Biological Stress

#### 2.1.1. Grazing Disturbance

Grazing causes changes in the species compositions and structures of original communities primarily by altering the strengths and directions of species interactions driven by environmental change. Shrub–grass interactions may be influenced by grazing intensity, livestock species, and plant palatability. The facilitation effect of shrubs on grass may show a monotonic increase or a unimodal increase following grazing, consistent with the prediction of the SGH. For example, the survival of a species that is characteristically a facilitation plant, *Caragana stenophylla* (Leguminosae), increased with grazing intensity in the Inner Mongolia Plateau region [12]. As grazing pressure increases, the role of shrubs in protecting seedlings from grass becomes more important. A study conducted in the Xilin River region of Inner Mongolia found that the facilitation effect of *Caragana stenophylla* on soil nutrients showed a unimodal trend along the grazing intensity gradient [13]. This ameliorative effect on nutrients may imply that the facilitation effect of shrubs on grasses also shows a unimodal trend with the grazing gradient. It is generally believed that overgrazing or selective foraging of grass species by livestock leads to a massive reduction in grass individuals and taxa. This elucidates their disadvantage in competing with shrubs, which gradually become the dominant grassland vegetation [14]. Shrubs with better palatability are also susceptible to livestock foraging and share part of the foraging pressure of grass species, and their competition with grass taxa is weakened, which means that appropriate grazing may slow down the process of shrub encroachment. Long-term livestock exclusion experiments in alpine grasslands on the Qinghai–Tibet Plateau showed that shrub encroachment induced by grazing exclusion was not only related to the elimination of direct interference from livestock but also to the competitive advantage of shrubs due to plant–plant interactions and the improvement of the deep soil resources of shrubs [12,15].

#### 2.1.2. Insects and Social, Burrowing, and Herbivorous Mammals

Social, burrowing, and herbivorous mammals are the key functional groups that shape grassland ecosystems, which helps maintain grasslands and open habitats [16]. For example, vizcachas, burrowing bettongs, and prairie dogs prevent invasion and establishment of shrubs through foraging and clipping [16,17]. Shrubland expansion in semi-arid regions of North America and Australia has been attributed, in part, to reductions in the populations of prairie dogs and bettongs, respectively [17,18]. Small burrowing mammals and megaherbivores can have mutualistic relationships. Grazing by megaherbivores facilitates increases in the population densities of burrowing mammal species that prefer more open grassland, thereby increasing their overall impact on a grassland ecosystem [16]. Many burrowing mammals preferentially forage on grasses, thereby facilitating the establishment of forbs [16,17]. However, some research has shown that rodent feeding, mowing, grass storage, and digging holes to create mounds reduce the aboveground and belowground biomass of herbaceous plants, thus weakening their competitiveness. Soil is pushed to the surface by wind and water erosion, which contributes to the degradation of grassland environments and is more conducive to the expansion of shrubs [19]. Some of the grassland rodents that feed on the fruits of leguminous shrubs can be used as vectors to assist in the dispersal of shrub seeds. Studies conducted in South Africa have found that rodents contribute to the spread of shrub seeds in grasslands, which plays an important role in grassland shrub encroachment [20].

Arthropods are important factors that alter the structures of plant communities. Invasions by exotic shrubs into habitats are associated with changes to insect communities [21]. Feeding by insect herbivores may have some effects, such as changing plant metabolism, hindering plant growth and reproduction, reducing plant community biomass, and changing plant community species diversity. In grassland ecosystems around the world, orthopterans including grasshoppers (Acrididae) and katydids/bush crickets (Tettigoniidae) are common and important herbivores [22,23]. Recent studies conducted in Europe and the continental United States found that plant biomass increases with grasshopper functional diversity [24,25]. This indicates that the effect of herbivore functional composition on grassland plant communities cannot be ignored. Insects may slow down grassland shrub encroachment to a certain extent by feeding on woody shrubs. Studies conducted in the Horqin Sandy Land found that seed pests reduced the competitiveness of *Caragana microphylla*, which affected the natural regeneration of its populations [26]. Similarly, it is speculated that some pests that cause shrub death or slow growth may also cause shrubs to occupy a weaker position in the competition with herbaceous species, which in turn slows shrub encroachment or promotes the succession of shrub-encroached grassland to grassland.

### 2.2. Abiotic Stress

#### 2.2.1. Shrub–Grass Interactions Under Drought Stress

The pattern of plant–plant interactions along stress gradients in semi-arid environments elucidated by Maestre et al. has been widely recognized [27]. As drought increases, the facilitation effect of shrubs on grasses may diminish or change to being competitive, thus contributing to the succession of grassland to shrub-encroached grassland. In southeastern Spain, the effects of *Retama sphaerocarpa* (a shrub) on grass biomass and species richness shifted from facilitation to competition as drought intensified [28]. A study conducted in an Oregon grassland found that the promoting effect of the shrub *Artemisia tridentata* on the reproductive potential of grasses increased then decreased along the drought gradient [29]. Weakening drought stress may help slow the process of grassland shrub encroachment. The research in the Inner Mongolian shrub-encroached grassland found that increased precipitation significantly increased the mean height, cover, and aboveground biomass of grasses, and as the degree of drought decreased, the relative cover of shrubs decreased while that of grasses increased, indicating that increased precipitation was beneficial to the growth of grass species but had no significant effect on shrub growth [30]. The reasons for this phenomenon may be that the maximum photosynthetic rate of the locally dominant herb *Leymus chinensis* is more sensitive to changes in precipitation and that its photosynthetic nitrogen use efficiency and water use efficiency are significantly higher than those of the invasive shrub *Caragana microphylla* under high soil moisture conditions. Most previous studies have centered on changes in indicator traits such as shrub-to-grass multiplicity, richness, density, biomass, survival, and reproductive rates along the drought gradient. From the perspective of leaf nutrients, the cumulative effects of *Caragana microphylla* on Ca, Cu, Fe, and Zn in leaves of plants from the non-grass functional group and P in leaves of plants from the grass functional group shifted from negative or neutral to positive with drought intensification in the Inner Mongolian grassland region [31]. This revealed drought effects on shrub–grass interactions in terms of deeper element utilization. 

#### 2.2.2. Shrub–Grass Interactions Under Temperature Stress

The argument that climate warming affects shrub–grass interactions arises from the observation that some encroaching shrub species are sensitive to low temperatures. For example, *Larrea tridentata* is prone to death from freezing-induced xylem cavitation [32]. Studies conducted in Spain have shown that due to rising temperatures, *Juniperus communis* has gradually replaced *Festuca aragonensis* and has become the dominant species. Shrubs have begun to expand to high altitudes and high latitudes [33]. There may be two explanations for the change in shrub–grass interaction caused by the temperature increase: (1) The degree of stress in suitable-temperature areas is elevated, and the competitive capacity of shrubs is increased. (2) Stresses decrease in low-temperature areas, as they are cooler. Thus, the effect of shrubs on grasses weakens. Liancourt et al., in the Ladakh region in India, found that the facilitation effect of the shrub *Caragana versicolor* on grasses increased then decreased with decreasing altitude (increasing temperature and drought) at the community level. There was an enhancing effect on the dominant companion grasses, *Elymus jacquemontii* and *Krascheninnikovia pungens*, whose presence and prevalence increased with decreasing elevation [34]. This research only focused on one shrub species, which ensured that the results were not affected by the facilitation of other shrub plants along the entire study gradient. It also suggested that the trend of plant interactions along the temperature gradient varied across study levels. Shrubs can alleviate the growth inhibition and distribution limitations of grass species at low temperatures. Amy et al. found that the promotion of the grass *Chamerion angustifolium* by the shrubs *Salix glauca* and *Salix brachycarpa* increased the grass’s winter survival and favored the expansion of its distribution range into high mountain areas where it otherwise would not survive [35].

#### 2.2.3. Shrub–Grass Interactions Under Salt Stress

Higher salt contents (e.g., Na^+^, K^+^, Cl^−^, and SO_4_^2−^) on vegetation patches beneath shrubs may interfere with the presence and conditions of grasses, thus modifying their spatial pattern and changing shrub–grass interactions [36]. Some salt-tolerant shrubs can alleviate a saline environment by reducing soil salinity and improving soil fertility, thus facilitating the survival and growth of grasses in the canopy. Rumbough found that the shrub *Atriplex canescens* ameliorated the physicochemical properties of saline soils, reduced their salinity, and increased the soil nutrient content, thus facilitating the growth of *Agropyron cristatum* and significantly increasing the grass production in the canopy [37]. Qi et al. studied the interactions between the shrub *Tamarix chinensis* and the grass species *Phragmites australis* and *Suaeda salsa* and found that, within the physiological limits of salt-tolerant plants, with the aggravation of salt stress, the plant interactions changed from competition to facilitation. When the salt stress exceeded the physiological tolerance of the salt-tolerant plants, the facilitation collapsed [38]. These results suggest that the changes in shrub–grass interactions along the salt stress gradient are in general agreement with the predictions of the SGH.

#### 2.2.4. Effects of Fire on Shrub–Grass Interactions

The degree to which fire affects shrub–grass interactions is related to the intensity and frequency of fires. Lower-frequency and -intensity fires are less damaging to shrubs than to grasses, making grasses less competitive. Studies conducted in the grassland of South Africa have found that small grassland fires are more conducive to shrub expansion. On the contrary, large fires are more conducive to the recovery of grass species [39]. The reasons for the suppression of shrub encroachment after a fire may include the following: (1) Compared with shrubs with deep roots and a long growth cycle, shallow-rooted grasses are more likely to obtain water and nutrients in the surface soil and have a stronger recovery ability in the short term. (2) Shrub seedlings are sensitive to fire, so severe fires have greater impacts on shrub plants. In the long-term process of community succession, other factors may lead to reductions in grasses and combustibles, thus reducing the intensity of fire disturbances, which is conducive to the expansion of shrubs and makes shrubs further replace the original grass plant community. In North America, as the degree of shrub encroachment is reduced, grass plants decrease, fire frequency decreases, and shrubs obtain a more stable living environment, further promoting shrub expansion, while at the same time the dominance of grasses and their biomass and cover decrease again, providing more favorable habitat conditions for shrubs and forming a positive feedback loop of shrub expansion [40]. Thus, fire management, controlling the frequency and sizes of fires, may affect the process of shrub encroachment. A fire plan can be formulated according to the degree of grassland shrub encroachment and the plant species, and periodic fire management can be carried out to control the number of shrubs and inhibit shrubs.

#### 2.2.5. Effects of Atmospheric CO_2_ Concentration on Shrub–Grass Interactions

An elevated atmospheric CO_2_ concentration may be a driver of grassland shrub encroachment. A 50-year experimental study conducted in the semi-arid grassland of Kruger National Park in South Africa found that with the passage of time, the increase in the CO_2_ concentration was the driving factor for the increase in shrub density in the region when other environmental driving factors such as rainfall remained unchanged [41]. On the one hand, studies have shown that an elevated CO_2_ concentration can enhance deep soil water storage. Compared with shallow-rooted grasses, shrubs with deep roots can obtain deep soil water [42]. On the other hand, shrubs and grasses have different physiological characteristics, and the differences in the respiratory responses between the two may lead to the expansion of shrubs. Most of the shrubs with increased abundance in shrub-encroached grasslands in tropical, subtropical, and temperate regions of North America are C_3_ species, while most of the replaced species are C_4_ species and grasses. Under low-CO_2_ conditions, the photosynthetic efficiency of C_4_ grasses is higher, while a high CO_2_ concentration can increase the net photosynthetic rate of C_3_ shrubs by inhibiting photorespiration. Therefore, the increase in the atmospheric CO_2_ concentration makes C_3_ shrubs more advantageous with respect to physiological activity, growth, and competitiveness [43,44]. In some dry cold desert steppes, even C_3_ grasses are replaced by C_3_ shrubs [45]. Therefore, the increase in the atmospheric CO_2_ concentration may not be the main factor driving changes in local vegetation.

#### 2.2.6. Species Specificity and Life-History Stages

The results of changes in plant–plant interactions with the degree of environmental stress are also related to the species specificity and life-history stages of the plants themselves. For example, shrubs provide some shade to grasses in the understory canopy, but the effect depends on the morphological characteristics of the shrub and the shade tolerance of the grass taxa. In the southwestern Iberian Peninsula, it was found that shrubs with scattered structures facilitated plant seedling survival and growth more than shrubs with clumped structures [46]. In the northern part of India, the shrub *Caragana versicolor* exerted a positive effect on erect-growing graminoid species while showing a negative effect on prostrate-growing grass species [47]. From the life-history point of view, during the seed germination stage, the facilitation of shrubs is mainly characterized by the provision of a microenvironment suitable for seed germination. After plant germination, shrubs can provide effective protection for seedlings, reducing their probability of being trampled and nibbled by phytophagous animals and reducing the negative effects of high temperatures and intense light on seedlings. As grasses grow, their competitive advantage related to water and other environmental resources increases and shrub–grass interactions are transformed from facilitation to competition. For example, in southeastern Spain, the facilitation effect of the shrub *Cistus clusii* on the grass *Stipa tenacissima* diminished as the grass matured, with *S. tenacissima* showing a neutral effect on the shrub during the seedling stage, then competition with the shrub after maturity, which was caused by the mature grass’s increased ability to access water [48].

## 3. Mechanisms of Shrub–Grass Interactions

### 3.1. Main Competition Mechanisms

The grass components of a plant community are influenced by a variety of mechanisms, such as allelopathy and competition for resources. Shrubs produce and release allelochemicals, which may hinder the growth and reproduction of grass species [49]. Shrubs and grasses compete for living space, light, water, nutrients, and other resources. This competition can lead to decreases in the species diversity of grass communities, expediting the expansion of shrubs and significantly altering the compositions and structures of the original grassland ecosystems.

#### Allelopathy and Competition

Allelopathy and competition possess different mechanisms. Chemically mediated plant–plant interactions are represented by allelopathy and allelobiosis [50]. By releasing toxic compounds (allelochemicals or signaling chemicals), a shrub may have a negative impact on the germination, growth, and survival of grasses. Competition is imposed by the environment, where plants draw from the same resource pools (i.e., shrubs consume water and nutrients and provide shade). Furthermore, allelopathy is a species-specific behavior, but competition occurs invariably during plant coexistence irrespective of the plant species. Importantly, competition occurs solely during the coexistence of living plants, but allelopathic potential can affect subsequent plant growth by the release of allelochemicals from deceased plants and their decomposing remnants. Plants biosynthesize a wide variety of allelochemicals, including terpenoids (monoterpenes, sesquiterpenes, diterpenes, and steroids), phenolics (simple phenolic acids, flavonoids, coumarins, and quinones), nitrogen-containing chemicals (alkaloids, benzoxazinoids, and cyanogenic glycosides), and other chemical families [51,52]. These allelochemicals are controlled by a range of genes, including those responsible for encoding transcription factors, which ultimately shape their allelopathic potentials [53]. In the Neotropical Savanna, leaf extracts from the shrub *Lepidaploa aurea* reduced the growth of other grasses [54]. Wagner et al., in an arid grassland dominated by perennial grasses, found that shrubs consistently competed with grasses for growth, and light-sensitive tufted grasses were affected by shrub shading, resulting in declining populations [55]. An overview of the types of shrub–grass interactions is shown in Table 1.

### 3.2. Main Facilitation Mechanisms

#### 3.2.1. Habitat Amelioration

The nurse effects provided by shrubs can help with the regeneration of native grasses by alleviating the detrimental effect that limits their growth and reproduction in degraded habitats. Shrubs can reduce the solar radiation reaching the understory or nearby grasses through their shading effect, can prevent or slow down the occurrence of plant photoinhibition, and can reduce the probability of high-temperature burns on leaves [56]. At the same time, the existence of shrubs is conducive to local maintenance and aggregation of water. On the one hand, shading can reduce temperatures, maintain higher soil humidity, and lower the transpiration of grasses. On the other hand, shading reduces the intensity of solar radiation and soil evaporation and maintains a high soil moisture content near the shrub. In desert environments, shrubs can protect and facilitate understory grasses by reducing the wind speed and flow abrasion. Studies have shown that *Nitraria tangutorum* shrubs play important roles in breaking the wind and sand fixation by reducing the air velocity to form shrub sand piles [57]. All the above processes increase the survival rates of grass species.

#### 3.2.2. Resource Enrichment

The precipitation interception capacity of shrub branches can increase the soil water content in shrubs, thus alleviating drought [58]. A study conducted in the Horqin Sandy Land of Inner Mongolia showed that the soil water content inside a *Caragana microphylla* shrub was higher than that outside the shrub [9]. In addition to live shrubs, after death the residual parts (branches, leaves, etc.) of shrubs can still play a role in increasing the soil water content and reducing the negative effects of high temperatures and water shortages on grass seedlings [59]. The hydraulic redistribution of shrub roots may also facilitate the growth of surrounding grass plants. Grass facilitation by woody plants through the process of hydraulic lift allows savannas to persist stably in mesic regions [60]. However, the process of hydraulic redistribution can result in hydraulic descent and hydraulic lift, and depending on inter-annual variations in precipitation. The redistribution of woody species can deplete or enhance soil moisture at graminoid rooting depths [61]. Hydraulic lift within overstory neighbors might be a more important driver of grass growth in drier regions where water is more limited or regions with pronounced dry seasons [62,63]. Shrub branches and leaves block and intercept large amounts of dust and litterfall so that more organic matter is deposited beneath the shrub canopy. To a certain extent, this increases the soil nutrient content in the shrub area. This phenomenon of significant accumulation of soil nutrients in local spaces caused by the formation of shrubs is called the fertile island effect (fertile islands). In a study conducted in northwestern Spain, it was found that the soluble nitrogen content, nitrification rate, NO concentration, and nitrogen absorption efficiency of plants beneath the shrub *Erica umbellata* were significantly higher than those outside the shrub [64].

#### 3.2.3. Protection Against Herbivory

Plant resistance to any biotic or abiotic stressor is generally assigned to two major categories: tolerance and avoidance traits [65,66,67]. Tolerance mechanisms increase the rate of leaf replacement through meristematic activity and several physiological processes, such as compensatory photosynthesis and increased carbon allocation to leaves [65,66,67]. Avoidance mechanisms minimize the frequency and/or intensity of herbivory by reducing plant palatability and accessibility [65,66,67]. Species that are grazed less severely (i.e., avoidance mechanisms), are capable of growing more rapidly following defoliation (i.e., tolerance mechanisms), or possess a combination of these two mechanisms realize a competitive advantage within a community [65,66,67]. Shrub avoidance mechanisms involve structural elements such as thorns, pubescence, raphides, and sclerophylly, all of which play important roles in protecting herbs from herbivores [68]. Chemical defenses such as the production of phenolics can deter herbivores from continued feeding following an initial bite [69]. Poor palatability or thorns on shrubs can protect herbaceous plants from herbivore predation. Studies conducted under grazing and fencing conditions have found that *Caragana intermedia* indirectly facilitates the growth of *L. chinensis* beneath shrubs as a ’herbivore shelter’ [70]. Palatability is multi-faceted. Reductions in palatability can be facultative and induced both in terms of tissue quality and structural defences or obligate and always expressed. These responses may reflect co-adaptive responses to the selective pressures of drought and grazing. Although both mechanisms are known to occur, how variations in climate drivers, as well as the intensity and duration of herbivory, affect the expression of these traits and subsequently modulate plant–plant interactions is not well understood.

#### 3.2.4. Introduction of Beneficial Organisms

Shrubs can indirectly facilitate grass species by introducing seed communicators [71], pollinators, mycorrhizal fungi [72], parasitic plants, and other beneficial soil microorganisms [73,74,75]. Shrub encroachment can lead to soil bacteria and fungal biomass increasing in shrub canopies compared to interspace grasses, and the composition of fungal communities has been observed to respond to shrub encroachment. For instance, arbuscular mycorrhizal fungi (AMF) can establish a symbiotic relationship with *Amorpha fruticosa*, a perennial leguminous shrub, forming arbuscular mycorrhiza (AM) [76]. Advances in molecular biology techniques now allow for quantitative analysis of protein expression differences during the colonization process of these symbiotic microorganisms, aiding in the understanding of plant interaction mechanisms. In one study, iTRAQ (Isobaric Tags for Relative and Absolute Quantification) combined with 2-D LC-MS/MS (two-dimensional liquid chromatography–tandem mass spectrometry) (GE Healthcare, Chicago, USA) was used to examine the expression of *A. fruticosa* mycorrhizal proteins at maturity [73]. The findings revealed that genes involved in plant metabolism and stress defense are crucial for the symbiotic relationship between AMF and *A. fruticose* [73]. Ericaceous shrubs can establish root–fungus associations with phosphate-solubilizing soil microorganisms (PSMs) involved in P-solubilization processes [77]. Fungal and actinomycete isolates obtained from a habitat of the shrub *Calluna vulgaris* can release soluble phosphate and exhibit diverse plant growth-promoting (PGP) activities [78]. The facilitation effect of soil available nitrogen fixed by legume shrubs on grasses is closely related to the symbiotic nitrogen fixation of rhizobia [74].

#### 3.2.5. Allelopathic Shrubs

It is commonly thought that allelopathic plants adversely affect the growth of nearby species. They can play a crucial role in determining the species diversity within a community by creating microenvironments where species interactions vary. For example, research conducted in the St. Martin de Londres valley in southern France revealed that the aromatic shrub *Thymus vulgaris* enhances species diversity both locally and at the community level [79]. This is achieved through the production of the monoterpene carvacrol, which creates a mosaic of thyme-modified and unmodified microhabitats differing in richness and species composition. Similar effects may be observed in other aromatic plants prevalent in Mediterranean ecosystems.

**Table 1:** Mechanisms of shrub–grass interaction.

Potential Mechanisms That May Occur Within Shrub–Grass Interactions		References
Habitat amelioration	Relieving solar radiation.	[56]
	Reducing soil salinity.	[36,37]
	Reducing wind erosion and water erosion.	[57]
	Enhancing the soil infiltration capacity.	[80]
	Providing soil humus.	[57]
Resource enrichment	Increasing the water content and nutrients in the soil in the microenvironment.	[9,58]
	Changing the microbial processes that affect soil carbon storage and nutrient cycling.	[81]
Association defense	Reducing eating and trampling by herbivores.	[70]
Introduction of beneficial organisms	Attracting pollinating animals and improving the pollination rate.	[82]
	Increasing soil microorganisms, such as mycorrhizal fungi, etc.	[8,55,72,73,83]
	Attracting seed-spreading animals (birds, etc.).	[71]
Competition for resources	Reducing soil moisture, reducing compaction, and enhancing aggregate stability.	[80]
	Different plant traits.	[84]
Allelopathy	Inhibitory effects of plants producing and releasing allelochemicals.	[49,53,54]

## 4. Effects of Shrub–Grass Interactions on Community Succession

Plant community succession is the result of synergistic interactions between a community’s internal relationships (species interactions and others) and external environmental factors. Shrub–grass interactions are one of the endogenous factors that drive grassland community succession and influence the direction of community succession. There are two possible outcomes due to changes in shrub–grass plant interactions after shrub encroachment. One is that shrubs can increase grass plant abundance, productivity, and ecosystem stability after shrub encroachment. The second is that the productivity and species diversity of grassland communities can be reduced, as an increase in shrub fertilization may cause the degradation of grassland ecosystems. The reasons for these results may be related to the types of plant species, the degree of shrub encroachment, climatic conditions, and other factors, so the impact of shrub fertilization on species composition has not yet been unanimously determined.

### 4.1. Effects of Shrub–Grass Interaction on Community Restoration Succession

The positive interaction between shrubs and grasses is one of the important mechanisms leading to community succession and driving community dynamics. This positive interaction may accelerate the restoration and succession of vegetation communities in degraded ecosystems by changing the spatial patterns of communities, which is more conducive to species coexistence, increasing community diversity and productivity. As nurse plants that can facilitate the survival and growth of other plant species under their canopies, shrubs are often used in the community restoration of degraded grasslands. At present, the facilitation of shrubs is often used to carry out ecological restoration research in deserts, alpine ecosystems, grasslands, and other harsh ecosystems. *Haloxylon ammodendron* is an effective tool for the restoration of arid desert vegetation, as it can reduce the wind erosion on the surfaces of dunes, provides protection for grasses, and effectively controls desertification [85]. Soil resources are enriched around the shrub *Salix cupularis* [86]. *Salix cupularis*, as a suitable pioneer shrub for vegetation restoration, contributes to the emergence of associated grasses in the desert alpine meadow of the Qinghai–Tibet Plateau [86]. In the typical steppe of Xilingol, Inner Mongolia, a study found that grassland with moderate shrub encroachment has higher species and functional diversity than grassland with mild or severe shrub encroachment, which means that a certain degree of shrub encroachment is beneficial for the maintenance of diversity and the restoration of degraded grassland [87]. The facilitation effect of grasses on shrubs can also accelerate the restoration of degraded grasslands. In the open grasslands of southeastern Spain, the survival rate of shrubs near *Stipa tenacissima* (grass) was significantly higher than that outside the grass, and *S. tenacissima* had a direct facilitation effect on the introduced shrubs [88]. This facilitation is of great significance for the effective restoration of degraded semi-arid ecosystems.

### 4.2. Effects of Shrub–Grass Interaction on Community Retrograde Succession

Shrub encroachment may be one of the causes of grassland ecosystem degradation. After grassland shrub encroachment, colonizing shrubs compete with the grass species that are the primary component of grassland habitats. The original uniformity of the grassland surface is destroyed, and wind and water erosion cause the deterioration of environmental conditions. Most shrub species have deep roots and strong drought resistance. Therefore, shrubs have higher viability and more competitive advantages than grasses in harsh environments. The survival of shrubs gradually changes the species compositions of plant communities, from grasses to shrub dominance. Knapp et al. found that the net primary productivity and species richness of grasses were significantly reduced by the competitive effect of shrubs compared with native communities at the scale of the North American steppe transect, thereby accelerating grassland degradation [89]. A study conducted in the New Mexico grasslands of the United States showed that the invasion of the shrub *Larrea tridentata* reduced the species diversity of grassland plant communities, and the stability of communities in shrub invasion areas decreased with an increase in shrub coverage [90]. Different functional groups of grass plants have different responses to the presence of shrubs. Zhang et al. found that shrub encroachment has a negative impact on the structure and function of grass plant communities [91]. Shrub invasion drives community succession by causing the extinction and colonization of non-random grass plant species. Low-lying grasses are more likely to become eradicated, while tall and shade-tolerant grasses benefit from shrubs. In the later stage of shrub invasion, there may be a high level of spatial heterogeneity of grassland soil nutrients and community composition. Xiong Xiaogang et al. divided the shrub invasion process into three relatively stable ecological states, namely native grassland, shrub grassland, and dune shrub encroachment [92]. After the emergence of shrub dunes, surface degradation is more serious. There are no shrubs in the surrounding area to block the strong wind and sand. The grasses are greatly reduced, and the function of the ecosystem tends to be lost. The dominance of shrubs in shrub–grass competition further aggravates grassland shrub encroachment, which provides environmental conditions for the invasion of shrubs. In Inner Mongolia, a study of different degrees of shrub grassland found that the soil nutrient content in the shrub *Caragana stenophylla* was significantly higher than that outside the shrub and gradually decreased when moving outward. This trend became more and more obvious with increases in the shrub degree [13]. The nutrient status of the soil around a shrub is more unfavorable to the survival of grasses outside the shrub. The soil outside the shrub is more likely to be eroded with the appearance of more bare land, eventually leading to the degradation of the grassland ecosystem. Considering the relationship between shrub–grass interactions, the degree of shrub encroachment, and the degradation of grassland ecosystems, controlling the number of shrubs and the degree of shrub encroachment may be an important measure to inhibit the degradation of shrub grassland.

## 5. Perspectives and Outlook

Shrub encroachment has become a widespread and increasing ecological phenomenon in grassland ecosystems around the world. Clarifying the factors that drive shrub encroachment and finding ways that the conversion of grassland to shrubland can be managed is currently an important research area. The role of shrub–grass interactions is clearly variable within the context of numerous environmental change drivers. At present, most of the research on shrub encroachment and shrub–grass interactions have been conducted through the observation and analysis of natural phenomena and processes, and there is a lack of research on their mechanisms. A material challenge for plant community ecology is determining how mathematical modeling could and should be applied to aid our understanding of the shrub–grass interaction mechanisms. This study summarizes and presents new information regarding the aboveground and underground parts of shrub and grass plants and the factors influencing their interactions. The other findings are as follows:

(1) The causes of shrub encroachment are complex, as there has been insufficient research on shrub encroachment caused by multi-factor coupling. It is necessary to study these factors and their combined effects on grassland shrub encroachment and shrub–grass interaction.

(2) From the perspective of the mechanism of the shrub–grass interaction, it is necessary to carry out more accurate simulation studies and establish corresponding models to clarify the phenomenon of grassland shrub encroachment caused by multiple mechanisms under different backgrounds. The influence of the indirect shrub–grass interaction mechanism on shrub encroachment needs to be further explored. Further assessments of allelopathy and allelobiosis in the context of environmental fluctuations and global shifts are needed. Future studies of the molecular pathways involved in allelopathy and allelobiosis in shrub–plant interactions, focusing on gene expression patterns, protein receptors, and their transport mechanisms are important research needs.

(3) Long-term, multi-dimensional monitoring and meta-analysis were conducted at a large scale to study the effects of shrub–grass interactions on community succession. The practical application of positive shrub–grass effects in a wide area needs to be further explored. It will be necessary to give full play to the positive effects of shrub–grass interactions on community succession to restore degraded grassland ecosystems.

(4) Although the SGH is indeed broadly tested in studies of plant community ecology, there is a lack of research on how the duration of an interaction affects the detected patterns, so we suggest this as a future research direction.
